# Expression of surfactant protein B is dependent on cell density in H441 lung epithelial cells

**DOI:** 10.1371/journal.pone.0184556

**Published:** 2017-09-14

**Authors:** Markus Fehrholz, Silvia Seidenspinner, Steffen Kunzmann

**Affiliations:** 1 University Children’s Hospital, University of Wuerzburg, Wuerzburg, Germany; 2 Clinic of Neonatology, Buergerhospital Frankfurt am Main, Frankfurt am Main, Germany; Emory University School of Medicine, UNITED STATES

## Abstract

**Background:**

Expression of surfactant protein (SP)-B, which assures the structural stability of the pulmonary surfactant film, is influenced by various stimuli, including glucocorticoids; however, the role that cell-cell contact plays in SP-B transcription remains unknown. The aim of the current study was to investigate the impact of cell-cell contact on SP-B mRNA and mature SP-B expression in the lung epithelial cell line H441.

**Methods:**

Different quantities of H441 cells per growth area were either left untreated or incubated with dexamethasone. The expression of SP-B, SP-B transcription factors, and tight junction proteins were determined by qPCR and immunoblotting. The influence of cell density on SP-B mRNA stability was investigated using the transcription inhibitor actinomycin D.

**Results:**

SP-B mRNA and mature SP-B expression levels were significantly elevated in untreated and dexamethasone-treated H441 cells with increasing cell density. High cell density as a sole stimulus was found to barely have an impact on SP-B transcription factor and tight junction mRNA levels, while its stimulatory ability on SP-B mRNA expression could be mimicked using SP-B-negative cells. SP-B mRNA stability was significantly increased in high-density cells, but not by dexamethasone alone.

**Conclusion:**

SP-B expression in H441 cells is dependent on cell-cell contact, which increases mRNA stability and thereby potentiates the glucocorticoid-mediated induction of transcription. Loss of cell integrity might contribute to reduced SP-B secretion in damaged lung cells via downregulation of SP-B transcription. Cell density-mediated effects should thus receive greater attention in future cell culture-based research.

## Introduction

The alveolar epithelium consists of a single cell layer formed by alveolar type I (ATI) and type II (ATII) cells, the latter deemed to be producers of pulmonary surfactant [1]. Surfactant protein (SP)-B ensures the mechanical functionality of the surfactant film [2]. Characterization of the factors affecting SP-B expression is considered of major clinical importance for maintaining or improving proper lung function [3].

To date, numerous regulators of SP transcription have been identified, including cell-cell and cell-matrix interactions, hormones, growth factors, inflammatory mediators, and agents that increase intracellular cyclic AMP levels [4]. Of the hormones identified, glucocorticoids are the main modulators of SP transcription in general and SP-B mRNA expression in particular [4]. Several transcription factors of the SP-B gene have been identified, of which thyroid transcription factor-1 (TTF-1) is recognized as the most prominent member [5]. Other transcription factors include specificity protein 1 (Sp1) and specificity protein 3 (Sp3), both of which are members of the hepatocyte nuclear factor 3 (HNF-3) family, as well as the neuregulin receptor erythroblastic leukemia viral oncogene homolog 4 (ErbB4) [6, 7]. Besides different transcription factors modifying SP-B expression, increased attention has been given to SP-B mRNA stability as a mechanism of post-transcriptional regulation following the suggestion that this greatly influenced the glucocorticoid-mediated increase of SP-B mRNA levels in lung epithelial cells [8].

To maintain alveolar cell layer integrity, ATI and ATII cell borders are sealed with junctional complexes [9], of which claudins-3, -4, -5, -7, and -18 are the most common tight junction proteins in airway epithelial cells [10].

Cell density-dependent regulation of gene expression has been extensively described in human and animal cell culture-based research [11–20] as well as for various cancer cell lines [21–30]. To the best of our knowledge, no such mechanism has been described for SPs in general or SP-B in particular. Disruption or injury of the epithelial cell layer may lead to airspace flooding and surfactant inactivation due to leaking plasma proteins [31]. To successfully treat pulmonary diseases, knowledge of the mechanisms mediating the formation and repair of the alveolar epithelial barrier and its integrity is mandatory [32].

If, and to what extent, the expression of SPs is linked to, or dependent on, an intact, united cell structure remains to be fully investigated. We hypothesized that cell-cell contact would have a considerable impact on the ability of ATII cells to support SP-B transcription and translation. The aim of our study was thus to identify the influence of cell density on SP-B expression in the absence or presence of dexamethasone, a representative glucocorticoid treatment. Glucocorticoids offer crucial stimulus during regular lung development and are used to accelerate fetal lung maturation when in threat of preterm birth. Loss of cell integrity may also potentially contribute to reduced secretion of SP-B in pulmonary diseases. Using increasing quantities of lung epithelial cells to simulate the varying integrity of uniform or mixed cell layers, we established that increased cell density influences SP-B mRNA stability, thereby affecting the overall transcriptional outcome of other stimuli such as glucocorticoids.

## Materials and methods

### Reagents, cells, and antibodies

Actinomycin D and dexamethasone were purchased from Sigma-Aldrich (St. Louis, CA).

Airway epithelial cells NCI-H441 (H441) (ATCC^®^ HTB­174^™^), a human lung adenocarcinoma cell line with characteristics of bronchiolar club epithelial cells [33], and A549 cells (ATCC^®^ CRM­CCL­185^™^) were both purchased from ATCC (LGC Standards, Teddington, UK). A549 cells were cultured in DMEM (Sigma Aldrich) supplemented with 10% fetal bovine serum (Gibco, Thermo Fisher Scientific, Waltham, MA), 100 U/mL penicillin, and 100 μg/mL streptomycin (Sigma Aldrich). H441 cells were cultured in RPMI 1640 (Sigma Aldrich) supplemented with 5% fetal bovine serum (Gibco), 100 U/mL penicillin, and 100 μg/mL streptomycin (Sigma Aldrich). Cells were incubated in their respective growth media at 37°C, in a humidified atmosphere with 5% CO_2_. For stimulation assays, cells were treated with 1 μM dexamethasone and/or 10 μg/mL actinomycin D.

Antibodies against SP-B were a generous gift from Dr. Jeffrey A. Whitsett, Perinatal Institute, Cincinnati Children’s Hospital Medical Center, Cincinnati, OH. Antibodies against β-actin (P/N 926–42212) and IRDye^®^ secondary antibodies were purchased from LI-COR Inc., Lincoln, NE. Polyclonal antibodies against claudin-5 and claudin-8 were from Abcam, Cambridge, UK. Horseradish peroxidase conjugated goat anti rabbit IgG was purchased from Novex^®^, Thermo Fisher Scientific. Horseradish peroxidase conjugated antibodies were visualized using Clarity Max^™^ Western ECL Substrate (Bio-Rad Laboratories, Hercules, CA).

### RNA extraction and RT-PCR

For RNA extraction, different H441 cell quantities were seeded on six well plates (Greiner, Frickenhausen, Germany) or cultured in 75-cm^2^ flasks (Greiner). Cells were washed with Dulbecco’s Phosphate Buffered Saline (DPBS; Sigma-Aldrich) and either left untreated, treated with dexamethasone, or with dexamethasone and actinomycin D. Following incubation at various time points, up to a maximum of 48 h, cells were washed again and total RNA was isolated using the NucleoSpin^®^ RNA Kit (Macherey-Nagel, Dueren, Germany) according to the manufacturer’s protocol. Total RNA was eluted in 60 μL nuclease-free H_2_O and stored at -80°C until reverse transcription. For total RNA quantification, a Qubit^®^ 2.0 Fluorometer (Thermo Fisher Scientific, Waltham, MA) was used as per the manufacturer’s recommendations. For RT-PCR, 0.3 to 1 μg of total RNA was reverse transcribed using the High-Capacity cDNA Reverse Transcription Kit (Thermo Fisher Scientific) according to the manufacturer’s instructions. First strand cDNA was diluted 1 to 10 with deionized, nuclease-free H_2_O (Sigma-Aldrich) and stored at -20°C for subsequent analysis.

### Real time quantitative RT-PCR (qPCR)

For mRNA transcript detection, 10 μL diluted first strand cDNA was analyzed in duplicates as previously described [34], using forward and reverse primers listed in Table 1.

**Table 1:** Primers for qPCR.

Gene symbol	Sequence accession #	Orientation	Sequence [5’ to 3’]	Amplicon length [bp]
B2M	NM_004048	forward	CCAGCAGAGAATGGAAAGTC	269
		reverse	GATGCTGCTTACATGTCTCG	
CAV1	NM_001753.4	forward	GATGACGTGGTCAAGATTGAC	186
		reverse	AGAGAATGGCGAAGTAAATGC	
CLDN1	NM_021101.4	forward	GCTTGGAAGACGATGAGG	170
		reverse	CCTGACCAAATTCGTACCTG	
CLDN2	NM_020384.3	forward	TGCCCTGTCTTCTAGATGCC	126
		reverse	CTCTTGCTCCTTGAACACCT	
CLDN3	NM_001306.3	forward	CACGCGAGAAGAAGTACAC	114
		reverse	TCTGTCCCTTAGACGTAGTCC	
CLDN4	NM_001305.4	forward	TAGCAAGAACAGAGTCCACCC	126
		reverse	CAGGCAGATCCCAAAGTCAG	
CLDN5	NM_003277.3	forward	GTCTTTACTCCATCGGCA	141
		reverse	CAGATTCTTAGCCTTCCCA	
CLDN7	NM_001307.5	forward	CATTAAGTATGAGTTTGGCCC	182
		reverse	AAGGAGATCCCAGGTCAC	
CLDN8	NM_199328.2	forward	CTTCTTTATCCTCTTCTCCCA	127
		reverse	CTTTCTTCTGAGTATAGCCCT	
ERBB4	NM_005235.2	forward	ACCCAAACAAGAATACCTG	144
		reverse	CTCATTCACATACTCATCCTC	
GAPDH	NM_002046.5	forward	CCATGGAGAAGGCTGGGG	195
		reverse	CAAAGTTGTCATGGATGACC	
FOXA1 (HNF3α)	NM_004496.3	forward	GGAACTGTGAAGATGGAAGGG	169
		reverse	ATGTTGCCGCTCGTAGTC	
OCLN	NM_002538.3	forward	CTTCCATTAACTTCGCCTG	200
		reverse	ATATTCCCTGATCCAGTCCT	
SP1	NM_138473.2	forward	GCTGTGGGAAAGTGTATGG	166
		reverse	GGCAAATTTCTTCTCACCTG	
SP3	NM_003111.4	forward	CTACCTTGAATACCAATGACC	141
		reverse	GTACCTCTTCCACCACCT	
TJP1 (ZO-1)	NM_003257.4	forward	CTTCCAGAACCAAAGCCT	190
		reverse	ATTCCAACATCATTTCCACC	
TTF1	NM_007344.3	forward	TAGCGAAGGAGATACTGAG	141
		reverse	ACCACGATTTCTTTGACTG	

A melt curve analysis was performed at the end of every run to verify the presence of single PCR products. In the case of SP-B and β-actin mRNA, specific probes (Thermo Fisher Scientific) were used as previously described [35]. mRNA transcript levels were normalized against those of β-actin or glyceraldehyde 3-phosphate dehydrogenase (GAPDH) and mean fold changes were calculated by the ΔΔC_T_ method of Livak and Schmittgen [36].

### Immunoblotting

Immunoblotting was performed as previously described [35]. Blots were probed with primary antibodies against SP-B, claudin-5, claudin-8, and β-actin overnight at 4°C, followed by staining with corresponding IRDye^®^ secondary antibodies (LI-COR Inc.) or an horseradish peroxidase conjugated goat anti rabbit IgG (Thermo Fisher Scientific) for 1 h at room temperature. Specific protein bands were visualized using an ODYSSEY^®^ Infrared Imaging System (LI-COR Inc.) or a ChemiDoc^™^ MP Imaging System (Bio-Rad Laboratories, Hercules, CA). Captured signals were quantified by densitometric analysis using Image Studio Lite v5.0.21 (LI-COR Inc.) or Image Lab^™^ Software v5.2.1 (Bio-Rad Laboratories).

### Statistical analysis

Results are given as mean ±standard deviation (SD). Unless otherwise stated, data were analyzed by one-way ANOVA with Bonferroni’s multiple comparison test. A *p*-value ≤ 0.05 was considered significant. All statistical analyses were performed using Prism^®^ version 6 (GraphPad Software, San Diego, CA).

## Results

### Impact of cell density on SP-B mRNA expression in H441 cells

**Fig 1:**
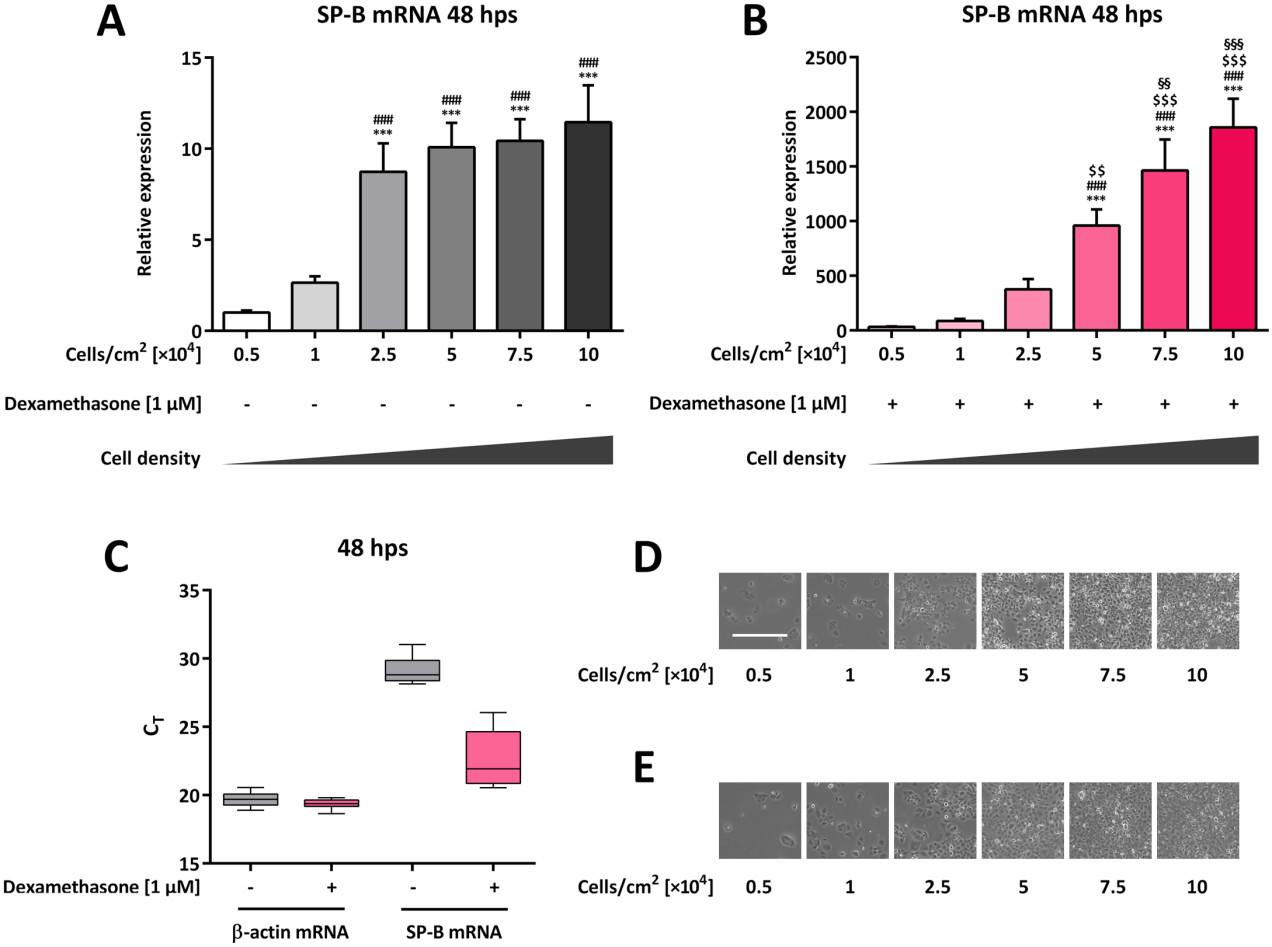
Impact of cell density on SP-B mRNA expression in H441 cells. Different quantities of H441 cells were seeded as indicated and either left untreated (**A**, **D**) or incubated with 1 μM dexamethasone for 48 h (**B**, **E**). SP-B mRNA levels were normalized to β-actin and fold differences compared to 0.5 × 10^4^ untreated cells per cm^2^ were calculated (**A**, **B**). Means +SD of n = 4 independent experiments are shown. (**C**) C_T_ range achieved for β-actin and SP-B mRNA combined for all different cell quantities with and without dexamethasone treatment. (**D**, **E**) Representative bright field images of H441 cells at different quantities without (**D**) and with (**E**) dexamethasone treatment. The bar in the upper left image represents 500 μm. *** *p* < 0.001 compared to 0.5 × 10^4^ cells per cm^2^, ### *p* < 0.001 compared to 1 × 10^4^ cells per cm^2^, $ $ *p* < 0.0 1 and $ $ $ *p* < 0.001 compared to 2.5 × 10^4^ cells per cm^2^, §§ *p* < 0.01 and §§§ *p* < 0.001 compared to 5 × 10^4^ cells per cm^2^.

To determine whether cell density affects SP-B mRNA abundance, different H441 cell quantities were cultured in the absence or presence of dexamethasone. SP-B mRNA levels were subsequently determined by qPCR. In comparison to that in untreated cells, dexamethasone treatment significantly increased SP-B mRNA levels for all cell densities tested (*p* < 0.001 for all samples); however, the dexamethasone-mediated fold-increase of SP-B mRNA levels varied widely in relation to cell quantity, ranging from 31.9 ±4.0-fold for 0.5 × 10^4^ cells per cm^2^, to 1,857 ±262-fold for 10 × 10^4^ cells per cm^2^ (combined comparison not shown). Comparison of SP-B mRNA abundance in 0.5 × 10^4^ and 1 × 10^4^ untreated cells per cm^2^ showed a gradual and significant increase, with highest levels observed for 10 × 10^4^ cells per cm^2^ (11.5 ±2.0-fold) (*p* < 0.0001 for all comparisons) (Fig 1A). When comparing dexamethasone-treated cells to each other, SP-B mRNA level increased significantly in both 5 × 10^4^ (*p* = 0.0023) and 7.5 × 10^4^ (*p* = 0.0097) cells per cm^2^ compared to the next lower cell quantity as well as compared to all other lower cell quantities (*p* < 0.0001) (Fig 1B). As evidenced by the C_T_ values, β-actin mRNA levels were less affected by different cell densities in untreated (19.7 ±0.5) and dexamethasone-treated cells (19.4 ±0.3), while the comparative range of SP-B mRNA levels was higher (29.2 ±1.0 and 22.6 ±1.9, respectively) (Fig 1C). β-actin was thus considered a suitable housekeeping gene for H441 cells of differing densities. In accordance with the increasing quantities of seeded cells, increasing cell-cell contact could be observed at higher densities (Fig 1D and 1E) with no alterations by the addition of dexamethasone (Fig 1E). At lower quantities, H441 cells appeared large in size possessing a large cytoplasm. With increasing density, H441 cells appeared smaller containing only little cytoplasm.

### Impact of cell density on mature SP-B expression in H441 cells

**Fig 2:**
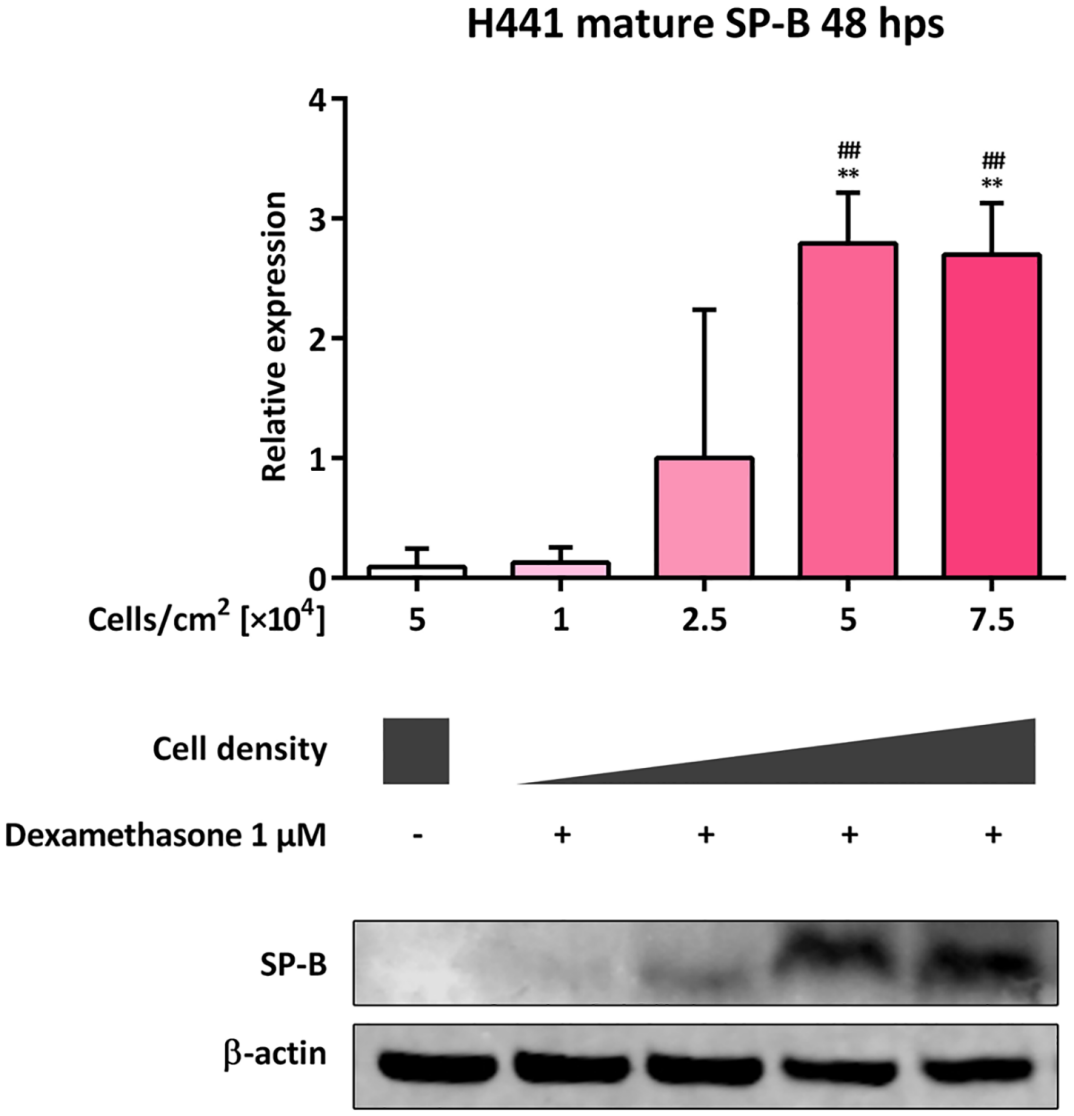
Impact of cell density on mature SP-B expression in H441 cells. Different quantities of H441 cells were seeded as indicated and either left untreated or incubated with 1 μM dexamethasone. After 48 h immunoblotting against SP-B and β-actin was performed. SP-B levels were normalized to β-actin and fold differences compared to 2.5 × 10^4^ dexamethasone-treated cells per cm^2^ were calculated. Means +SD of n = 3 independent experiments are shown. ** *p* < 0.01 compared to 5 × 10^4^ untreated cells per cm^2^, ## *p* < 0.01 compared to 1 × 10^4^ dexamethasone-treated cells per cm^2^.

We next measured mature SP-B (8–9 kDa) levels in lysates of dexamethasone-treated cells, seeded at different densities, after 48 h by immunoblotting. Negligible levels of mature SP-B were detected in 5 × 10^4^ untreated and 1 × 10^4^ dexamethasone-treated cells per cm^2^. Mature SP-B expression levels were significantly increased in 5 × 10^4^ and 7.5 × 10^4^ dexamethasone-treated cells per cm^2^, in comparison to that in 5 × 10^4^ untreated (*p* = 0.0034 and *p* = 0.0045, respectively) and 1 × 10^4^ dexamethasone-treated cells per cm^2^ (*p* = 0.0038 and *p* = 0.0050, respectively) (Fig 2).

### Impact of cell density on the expression of SP-B transcription factor mRNAs in H441 cells

**Fig 3:**
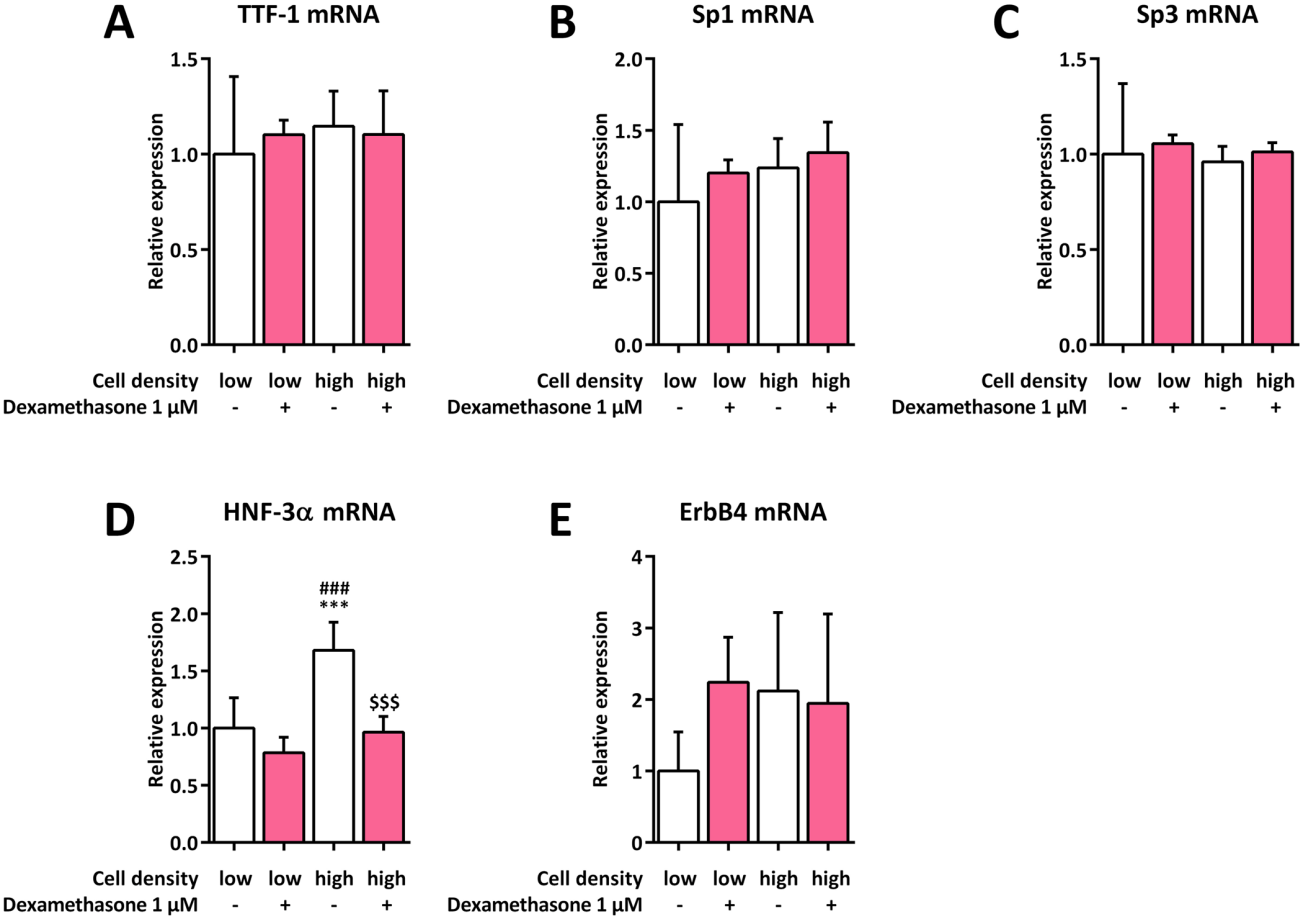
Impact of cell density on the expression of SP-B transcription factor mRNAs in H441 cells. H441 cells were seeded at low (1 × 10^4^ cells per cm^2^) and high (7.5 × 10^4^ cells per cm^2^) densities and either left untreated or incubated with 1 μM dexamethasone for 24 h. qPCR of TTF-1 (**A**), Sp1 (**B**), Sp3 (**C**), HNF-3α (**D**), and ErbB4 mRNA (**E**) was performed, mRNA levels were normalized to those of GAPDH, and fold differences compared to 1 × 10^4^ untreated cells per cm^2^ were calculated. Means +SD of n = 5 experiments are shown. *** *p* < 0.001 compared to 1 × 10^4^ untreated cells per cm^2^, ### *p* < 0.001 compared to 1 × 10^4^ dexamethasone-treated cells per cm^2^, $ $ $ *p* < 0.001 compared to 7.5 × 10^4^ untreated cells per cm^2^.

To investigate whether cell density affects the expression of various SP-B transcription factor mRNAs, low and high H441 cell quantities were cultured in the absence or presence of dexamethasone. SP-B transcription factor mRNAs were subsequently quantified by qPCR. Neither cell density nor dexamethasone treatment had any influence on TTF-1, Sp1 or Sp-3 mRNA expression (Fig 3A–3C). HNF-3α mRNA expression only increased significantly in high-density untreated cells (1.7 ±0.2-fold, *p* = 0.0005), with dexamethasone treatment preventing this induction (*p* = 0.0003) (Fig 3D). ErbB4 mRNA expression was slightly increased in all groups compared to that in low-density untreated cells, although this was not statistically significant (Fig 3E).

### SP-B mRNA expression in H441 cells co-cultured with A549 cells at different cell densities

**Fig 4:**
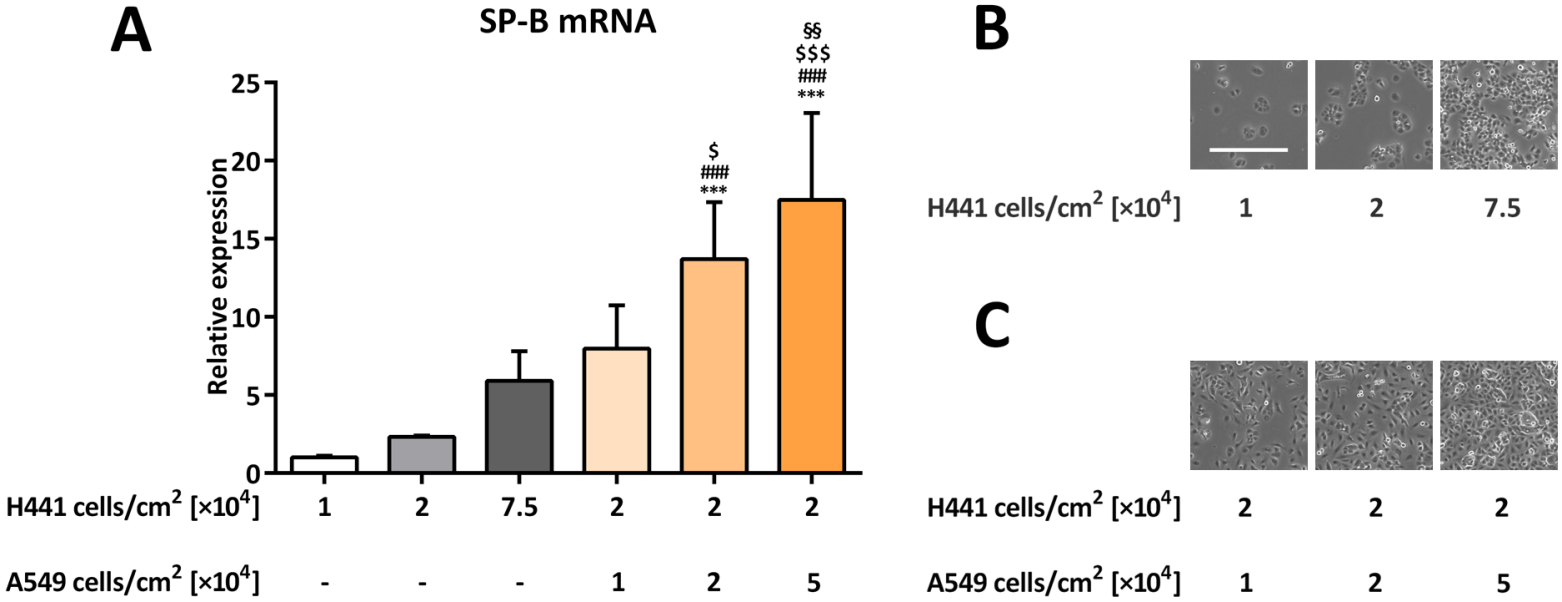
SP-B mRNA expression in H441 cells co-cultured with A549 cells at different cell densities. Different quantities of H441 cells were seeded alone or co-cultured with different quantities of A549 cells as indicated for 48 h. (**A**) SP-B mRNA levels were normalized to β-actin and fold differences compared to 1 × 10^4^ H441 cells per cm^2^ without A549 cells were calculated. Means +SD of n = 4 independent experiments are shown. (**B**, **C**) Representative bright field images of H441 cells alone (**B**) or co-cultured with A549 cells (**C**). The bar in the upper left image represents 500 μm. *** *p* < 0.001 compared to 1 × 10^4^ H441 cells per cm^2^ without A549 cells, ### *p* < 0.001 compared to 2 × 10^4^ H441 cells per cm^2^ without A549 cells, $ *p* < 0.05 and $ $ $ *p* < 0.001 compared to 7.5 × 10^4^ H441 cells per cm^2^ without A549 cells, §§ *p* < 0.01 compared to 2 × 10^4^ H441 cells per cm^2^ co-cultured with 1 × 10^4^ A549 cells per cm^2^.

To establish whether cells with low or nonexistent SP-B expression levels may initiate SP-B mRNA transcription in H441 cells via cell-cell contact, we incubated H441 cells with increasing amounts of A549 cells, which express low to nonexistent levels of SP-B, including its mRNA [35]. A549 cells are therefore considered a suitable ATI model in terms of its SP-B expression. In comparison to that in 2 × 10^4^ pure H441 cells per cm^2^, SP-B mRNA expression was significantly increased in 2 × 10^4^ H441 cells per cm^2^ co-cultivated with either 2 × 10^4^ (13.7 ±3.6-fold) or 5 × 10^4^ (17.5 ±5.6-fold) A549 cells per cm^2^ (*p* = 0.0007 and *p* < 0.0001, respectively) (Fig 4A). In contrast to H441 cells (Figs 1D, 1E and 4B), higher cell density had less influence on the phenotype of A549 cells (Fig 4C).

### Impact of cell density on mRNA expression of tight junction or tight junction-related proteins in H441 cells

**Fig 5:**
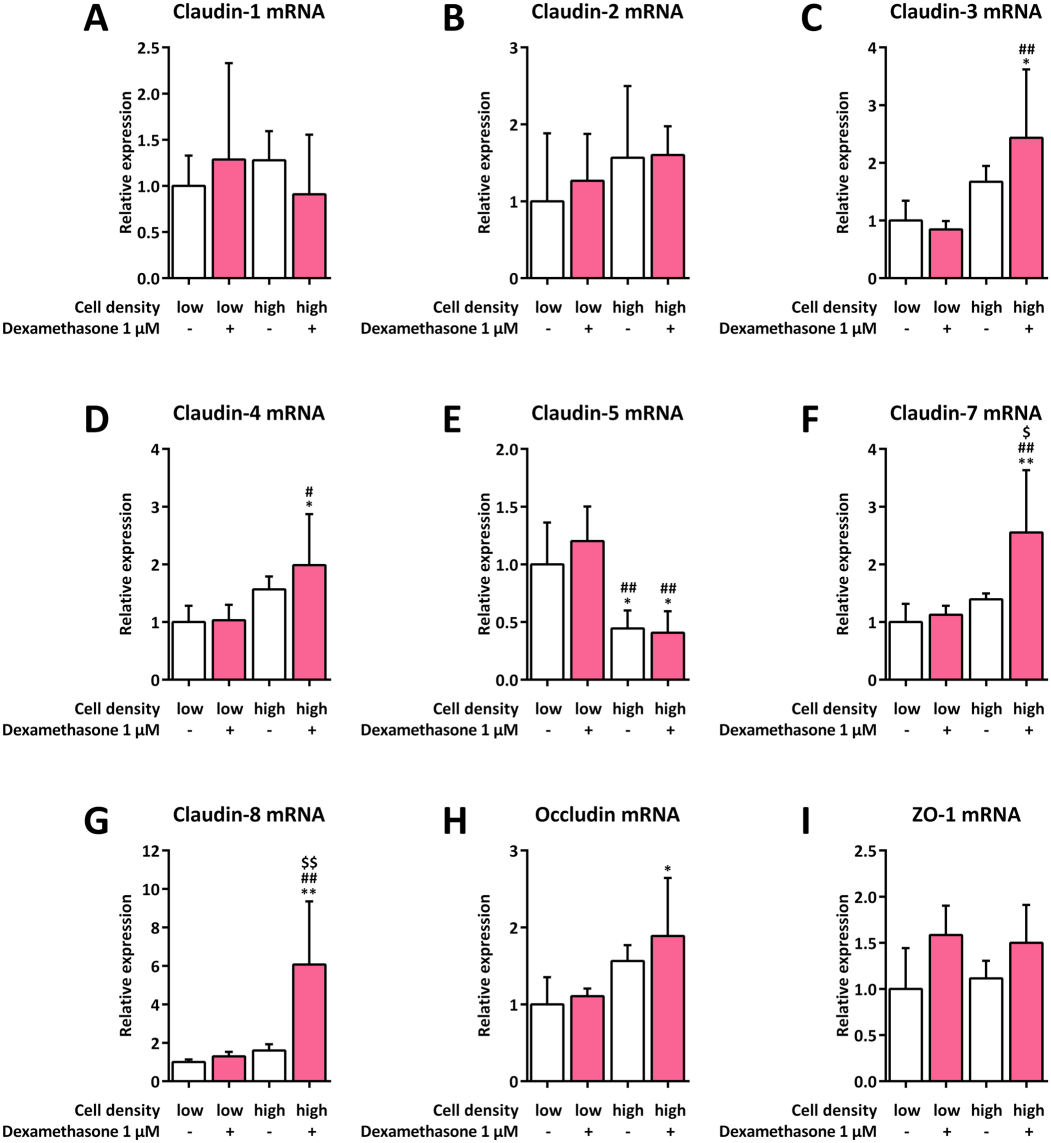
Influence of cell density on mRNA expression of tight junction or tight junction-related proteins in H441 cells. H441 cells were seeded at low (1 × 10^4^ cells per cm^2^) and high (7.5 × 10^4^ cells per cm^2^) densities and either left untreated or incubated with 1 μM dexamethasone for 24 h. qPCR of claudin-1 (**A**), claudin-2 (**B**), claudin-3 (**C**), claudin-4 (**D**), claudin-5 (**E**), claudin-7 (**F**), claudin-8 (**G**), occludin (**H**), and ZO-1 mRNA (**I**) was performed, mRNA levels were normalized to those of GAPDH, and fold differences compared to 1 × 10^4^ untreated cells per cm^2^ were calculated. Means +SD of n = 5 experiments are shown. * *p* < 0.05 and ** *p* < 0.01 compared to 1 × 10^4^ untreated cells per cm^2^, # *p* < 0.05 and ## *p* < 0.01 compared to 1 × 10^4^ dexamethasone-treated cells per cm^2^, $ *p* < 0.05 and $ $ *p* < 0.01 compared to 7.5 × 10^4^ untreated cells per cm^2^.

To analyze whether cell density impacts mRNA expression for various tight junction or tight junction-related proteins potentially involved in initiation of SP-B mRNA transcription, H441 cells were seeded at low and high densities and cultured in the absence or presence of dexamethasone. We found claudin-18 mRNA level to be beyond the detection limit in all experimental conditions assessed in this study. Neither cell density nor dexamethasone treatment had any influence on claudin-1, claudin-2, or zonula occludens-1 (ZO-1) mRNA expression (Fig 5A, 5B and 5I). A significant increase in mRNA expression was only observed for the combination of high cell density with dexamethasone treatment for claudin-3 (2.4 ±1.2-fold, *p* = 0.0155), claudin-4 (2.0 ±0.9-fold, *p* = 0.0379), claudin-7 (2.6 ±1.1-fold, *p* = 0.0033), claudin-8 (6.1 ±3.3-fold, *p* = 0.0011), and occludin (1.9 ±0.8-fold, *p* = 0.0298) (Fig 5C, 5D and 5F–5H). A significant reduction of claudin-5 mRNA expression was observed for high cell densities in both untreated (0.4 ±0.2-fold, *p* = 0.0263) and dexamethasone-treated cells (0.4 ±0.2-fold, *p* = 0.0165) (Fig 5E).

### Impact of cell density on claudin-5 and claudin-8 expression in H441 cells

**Fig 6:**
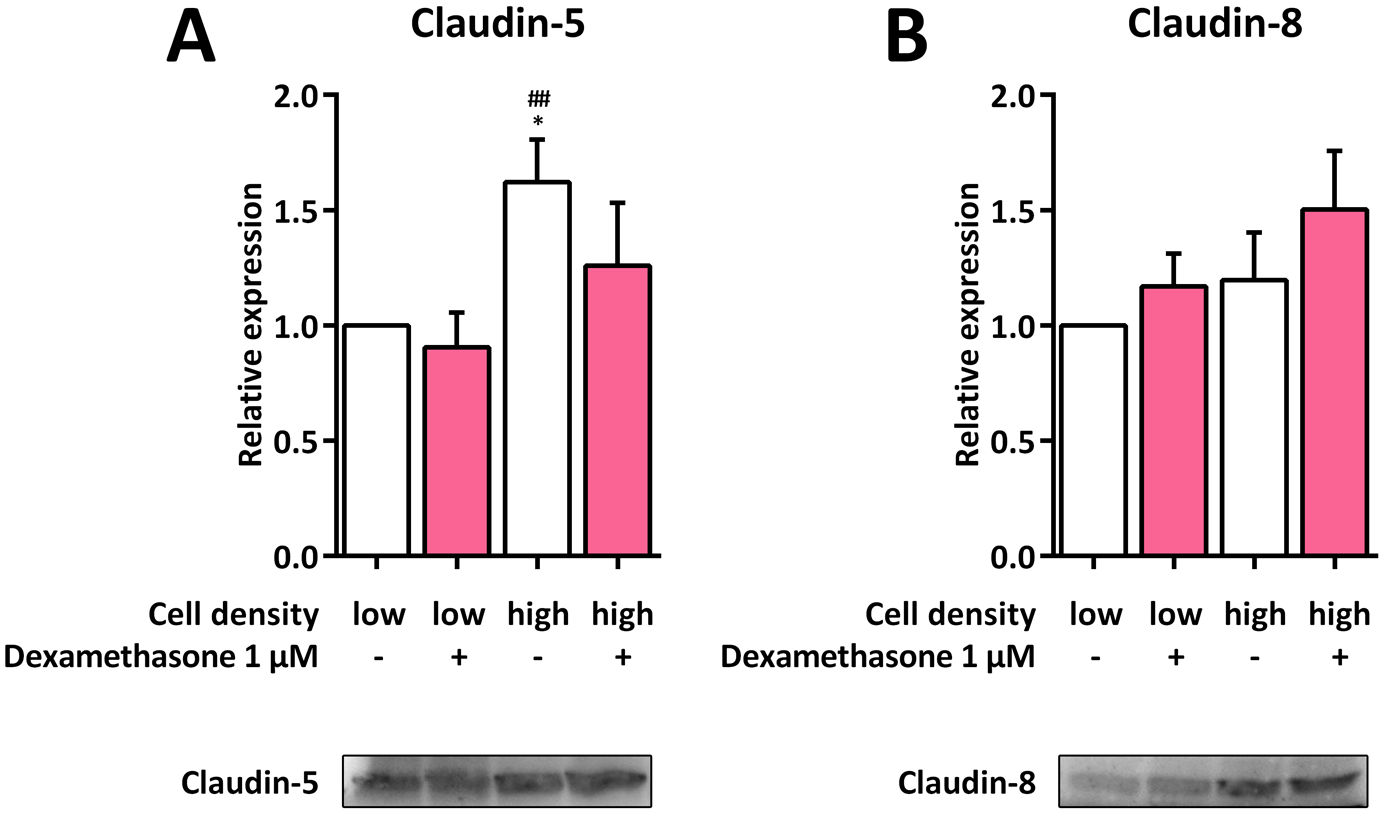
Immunoblots against claudin-5 and claudin-8 from H441 cells at different densities. H441 cells were seeded at low (1 × 10^4^ cells per cm^2^) and high (7.5 × 10^4^ cells per cm^2^) quantities and either left untreated or incubated with 1 μM dexamethasone for 48 h. Immunoblots against claudin-5 (**A**) and claudin-8 (**B**) were performed, protein levels were normalized to total blotted protein, and fold differences compared to 1 × 10^4^ untreated cells per cm^2^ were calculated. Means +SD of n = 3 independent experiments are shown. * *p* < 0.05 compared to 1 × 10^4^ untreated cells per cm^2^, ## *p* < 0.01 compared to 1 × 10^4^ dexamethasone-treated cells per cm^2^.

To analyze whether the upregulation of claudin-8 mRNA and the downregulation of claudin-5 mRNA, both of which were influenced the most by higher cell densities, was also reflected by alterations of the respective protein level, we performed immunoblots of H441 cells seeded at low and high densities and cultured in the absence or presence of dexamethasone. However in contrast to the transcriptional level, claudin-5 was upregulated in high-density cells without dexamethasone compared to low-density cells without dexamethasone, while no alterations could be observed for all other groups. Neither cell density nor dexamethasone treatment led to a significant regulation of claudin-8 (Fig 6).

### Influence of cell density on SP-B mRNA stability in H441 cells

**Fig 7:**
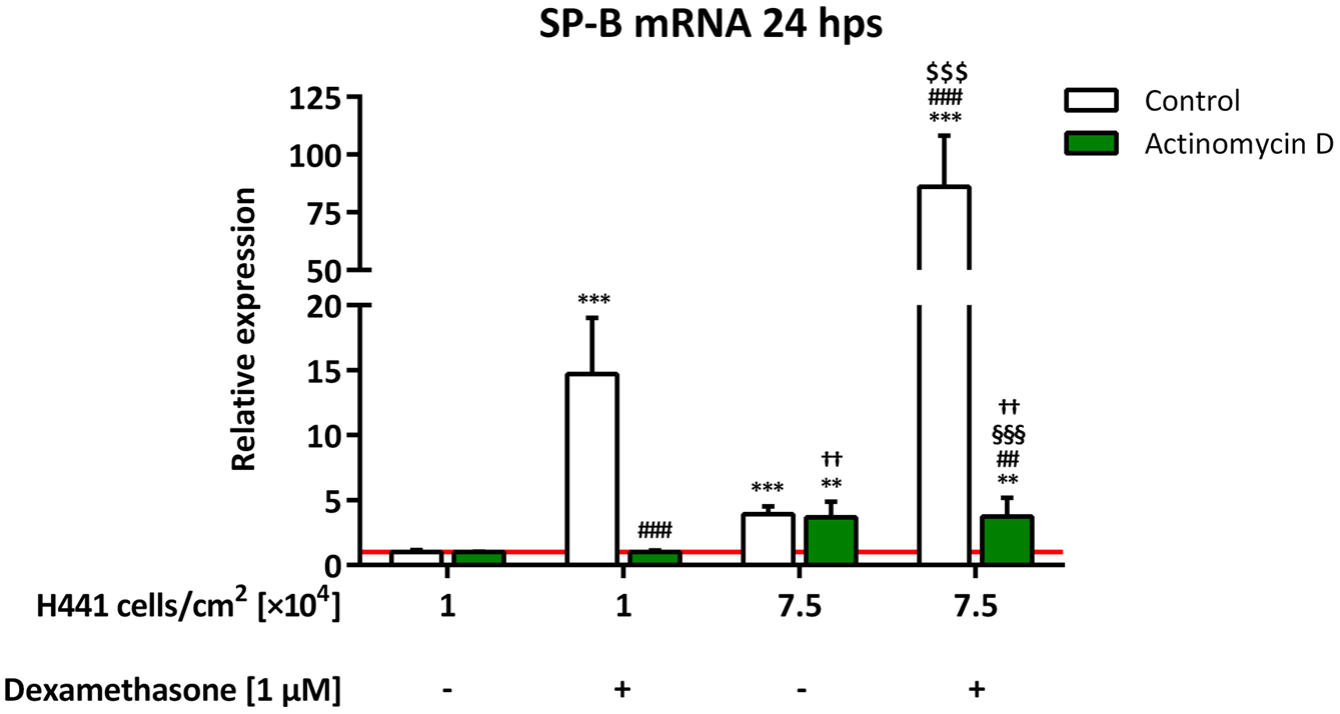
Influence of cell density on SP-B mRNA stability in H441 cells. Different quantities of H441 cells were seeded as indicated and either left untreated or incubated with 1 μM dexamethasone and/or 10 μg/mL actinomycin D for 24 h. SP-B mRNA levels were normalized to β-actin and fold differences compared to 1 × 10^4^ untreated cells per cm^2^ were calculated for each group. Means +SD of n = 4 experiments are shown. Statistical analyses were performed using Student’s unpaired *t* test and only relevant significances are shown. ** *p* < 0.01 and *** *p* < 0.001 compared to 1 × 10^4^ untreated cells per cm^2^, ## *p* < 0.01 and ### *p* < 0.001 compared to 1 × 10^4^ dexamethasone-treated cells per cm^2^, $ $ $ *p* < 0.001 compared to 7.5 × 10^4^ untreated cells per cm^2^, §§§ *p* < 0.001 compared to 7.5 × 10^4^ dexamethasone-treated cells per cm^2^, †† *p* < 0.01 compared to 1 × 10^4^ dexamethasone- and actinomycin D-treated cells per cm^2^.

To establish whether increased mRNA stability is responsible for the observed cell density-dependent elevation of SP-B mRNA, we treated low and high H441 cell quantities with dexamethasone and/or the transcription inhibitor actinomycin D. Of note, only high cell densities influenced SP-B mRNA abundance in the presence of actinomycin D (Fig 7). SP-B mRNA levels were not significantly different between 1 × 10^4^ cells per cm^2^ treated with or without dexamethasone (1.0 ±0.1-fold for both), as well as between 7.5 × 10^4^ cells per cm^2^ treated with or without dexamethasone (3.8 ±1.4-fold and 3.7 ±1.2-fold, respectively), in the presence of actinomycin D. SP-B mRNA expression in 7.5 × 10^4^ cells per cm^2^ (3.8 ±1.4-fold) treated with actinomycin D and dexamethasone was thus significantly lower than that in 1 × 10^4^ cells per cm^2^ (*p* = 0.0030) and 7.5 × 10^4^ cells per cm^2^ (*p* = 0.0003) treated with dexamethasone without actinomycin D.

## Discussion

In this study, we established that SP-B transcription in H441 cells is dependent on cell-cell contact. Although cell-cell contact has a relatively small impact as a sole stimulus, increased cell-cell contact of H441 cells was able to significantly potentiate other transcription-initiating stimuli like dexamethasone, thereby influencing SP-B expression. In addition to a direct effect on SP-B expression *in vivo*, a loss of alveolar epithelial cell integrity might affect glucocorticoid-initiated surfactant production to an even greater extent in injured alveolar epithelial cell layers.

Cell density-dependent expression is already known for a large number of genes and a wide diversity of cell types, including leukocytes [11], and stromal, smooth muscle, liver, and mesenchymal stem cells [15–20]. Our findings confirm the existence of cell density-dependent effects for SP-B expression in H441 cells *in vitro*. This regulatory mechanism is suggested to have a large impact on the glucocorticoid-mediated induction of SP-B mRNA expression and therefore, also possibly on other glucocorticoid-regulated genes in lung epithelial cells. The latter assumption is in accordance with the cell density-dependent modulation of glucocorticoid-induced gene expression in hepatoma cells [21]. In contrast, β-actin mRNA expression was barely affected, if at all, by differing cell densities, justifying its use as a housekeeping gene in our experimental setting. With regard to the potential importance of cell density for the expression of SPs, the lack of SP-B expression in A549 cells might correlate with their inability to form functional tight junctions and therefore tight monolayers of polarized cells [37].

To establish the underlying mechanism of the observed cell density-mediated increase of SP-B expression, we first focused on a potential alteration in the expression of known SP-B transcription factors. We found HNF3α transcription factor mRNA to be significantly upregulated in high-density cells which was however prevented by the additional treatment with dexamethasone. Thus, HNF3α is not likely to be responsible for the further increase of SP-B after a combination of these two stimuli. Although ErbB4 mRNA expression was slightly higher in all other groups compared to that in low-density untreated cells, this finding was not statistically significant and thus indicates no predominant role for ErbB4 with regard to the observed effect either.

In lung alveoli, cubic, surfactant-producing ATII cells are surrounded by surfactant-negative ATI cells. Although virtually equal in number, the large area ATI cells occupy is a much larger surface than that covered by ATII cells [38], which rarely allows ATII cells to come into close contact with one another. By co-culturing H441 with A549 cells, the latter expressing significantly less SP-B mRNA [35] and therefore a suitable model for SP-B-negative ATI cells in our setting, we found that even non-specific cell-cell contact was able to facilitate increased SP-B mRNA expression. This indicates that it is not the type of cell that is important, but rather the mechanisms induced by the non-specific cell-cell contact that occurs via adjacent membranes that is of importance.

We thus next investigated whether the observed increase in SP-B expression could be directly linked to an altered expression of a specific tight junction protein in lung epithelial cells, which are important for regulating permeability in confluent cell layers [10]. Apart from downregulated claudin-5 mRNA expression, which could not be observed on the protein level, high cell density did not influence tight junction markers or tight junction-related proteins as a sole stimulus, suggesting that these proteins are likely to not be involved in the signal cascades that initiate the cell density-dependent regulation of SP-B expression. Claudin-5, however, has been shown to increase permeability in human airways *in vivo* [39]. Hence, the observed downregulation of claudin-5 mRNA expression at higher cell densities may indicate a simultaneous decrease in permeability, which correlates with the observation that the barrier function of primary human fetal lung cells increases at confluency [40]. Nevertheless, as expression of claudin-5 protein did not correlate with that of its mRNA in our setting, this conclusion is not necessarily valid.

Regulation of SP-B expression is not only based on transcriptional modification but is also related to an alteration of SP-B mRNA stability [41]. Our data clearly show that, at least for H441 cells, increased cell density has a minor but prominent impact on SP-B mRNA stability, thereby markedly affecting the transcriptional outcome of other stimuli such as glucocorticoids. In contrast to work describing dexamethasone-related effects on SP-B mRNA stability [8, 41, 42], the results of our experiments, using actinomycin D as an inhibitor of *de novo* mRNA synthesis, indicate that dexamethasone had no effect on SP-B mRNA stability independent of *de novo* gene transcription. SP-B mRNA levels in high-density cells were found to be identically higher in comparison to low-density cells after actinomycin D treatment, irrespective of whether or not they were treated with dexamethasone. A dexamethasone-mediated increase of mRNA stability independent of *de novo* gene transcription would also have affected SP-B mRNA levels in the presence of the transcriptional inhibitor, as seen for the cell-cell contact stimulus, which was not observed. This is in contrast to work on human lung explants [43], which demonstrated an increased SP-B mRNA half-life in the presence of dexamethasone. A possible explanation for this discrepancy might be that in our experimental setting, dexamethasone and actinomycin D treatment were conducted simultaneously, whereas the other groups employed a pre-incubation step with dexamethasone [8, 41–43]. Increased confluency in samples pretreated with dexamethasone might have increased the stability of SP-B mRNA *per se*, leading to the divergent results. This would also help explain the reported dexamethasone-related effects observed in the absence of the glucocorticoid-receptor [8].

The results described in this study are subject to certain limitations. First, to overcome the very restricted proliferation profile of primary lung epithelial cells in culture [37], the papillary adenocarcinoma cell line H441 was used, which might reflect an incomplete *in vivo* situation. Second, cell-density effects on stretch-dependent modifications of SP-B transcription [44] may be important for the observed effects. Last, actinomycin D may introduce significant changes in cell physiology which additionally affect SP-B mRNA stability [45].

H441 was the only available cell line with sufficient *in vitro* levels of SP-B expression for this study; however, further work should focus on the underlying mechanisms for the cell density-dependent expression of surfactant genes in primary lung epithelial cells. The additional regulatory mechanism for SP-B expression revealed here may be significant *in vivo* with respect to alterations of alveolar epithelial integrity, e.g., by inflammation or ventilation. Moreover, these alterations of integrity may greatly impair the stimulatory abilities of glucocorticoids. Both should also be addressed in future studies.

### Conclusion

Induction of SP-B expression in lung epithelial cells is complex and includes various factors such as cell-cell contact and mRNA stability. Initiation of *de novo* SP-B mRNA synthesis by glucocorticoids is likely to be independent of cell-cell contact, while SP-B mRNA stability is markedly increased at higher cell densities, thereby potentiating dexamethasone-mediated SP-B transcription. Loss of cell integrity might contribute to reduced SP-B secretion in damaged lung cells via downregulation of SP-B transcription. Further, it was established that providing an intact, united cell structure is necessary to simulate the *in vivo* conditions for SP-B expression. In conclusion, cell density-mediated effects should receive greater attention in future cell culture-based research.
